# Transcriptional Profiling of Caudal Fin Regeneration in Zebrafish

**DOI:** 10.1100/tsw.2006.326

**Published:** 2006-06-02

**Authors:** Michael Schebesta, Ching-Ling Lien, Felix B. Engel, Mark T. Keating

**Affiliations:** Howard Hughes Medical Institute, Department of Cardiology and Department of Pediatrics, Children's Hospital, Department of Cell Biology, Harvard Medical School, 320 Longwood Avenue, Boston, MA 02115, USA

**Keywords:** regeneration, fin, zebrafish, microarray, bmp, bambi, dlx, her

## Abstract

Regeneration of severed limbs in adult animals is restricted to urodele amphibians. Mammals, including humans, have very limited regenerative capabilities and even with proper treatment, only the tips of our digits can grow back. Teleost fish can regenerate amputated fins, the evolutionary ancestors of limbs. To elucidate the principles of limb-fin regeneration, we performed an Affymetrix microarray screen on regenerating caudal fins 12, 24, 48, and 72 h post amputation. Approximately 15,000 zebrafish transcripts were analyzed, identifying 829 transcripts as differentially expressed during regeneration. Of those, 563 were up-regulated and 266 were down-regulated. We constructed a comprehensive database containing expression data, functional assignment, and background information from the literature for each differentially expressed transcript. In order to validate our findings, we employed three approaches: (1) microarray expression analysis of genes previously implicated in fin regeneration, (2) RT-PCR analysis of genes newly identified as differentially expressed during regeneration, and (3) *in situ hybridization* of the up-regulated genes bambi, *dlx5A*, and her6. Moreover, we show that Smad 1/5/8 proteins, effector molecules of Bmp signaling, are phosphorylated during fin regeneration. Taken together, we provide a comprehensive database of fin regeneration that will serve as an important tool for understanding the molecular mechanisms of regeneration.

### INTRODUCTION

Urodele amphibians, such as newt and axolotl, faithfully replace a lost limb over the course of 1–2 months[1,2]. In contrast, mammals show very limited regenerative capability in response to the severance of a limb[3]. Humans can regrow digit tips with proper treatment, but only the very distal part of the finger or toe[4,5,6]. To better understand why certain animals can regenerate their limbs while others cannot, a more comprehensive knowledge of this process needs to be elucidated. Once the molecular mechanisms of limb regeneration are known, we may consider applying this knowledge to improve the regenerative capacity of mammals, humans in particular.

Teleost fish are also capable of regenerating multiple complex body structures, including the fin[7,8,9]. The zebrafish (*Danio rerio*), a teleost fish, has been extensively studied during embryonic development. In contrast to newt and axolotl, zebrafish are easily maintained in high numbers and small spaces. Furthermore, multiple genetic and molecular tools have been developed for zebrafish. The zebrafish tail (caudal) fin regenerates over the course of 1–2 weeks, as opposed to 1–2 months for urodele limbs. Because the fin is the primordial progenitor of the limb, it contains many tissues similar to the limb. Zebrafish fins are comprised of epithelium, mesenchyme, bony fin rays, cartilage, blood vessels, nerves, connective tissue, and pigment[7]. One difference to vertebrate limbs is the absence of skeletal muscle tissue in the fin. Because of technical and practical advantages, we selected zebrafish fin regeneration as our model system for limb regeneration.

Fin regeneration can be divided into three phases: wound healing, blastema formation, and regenerative outgrowth[7]. During the first 12 hours post amputation (hpa), migrating epidermal cells close the wound. This initial phase of wound healing is independent of proliferation[10]. Over time, the wound epithelium builds up multiple layers and forms the wound epidermis or apical epidermal cap (AEC)[11]. The AEC is believed to fulfill a similar essential role as the apical ectodermal ridge (AER) during development, secreting patterning factors for the developing limb[12]. Genes of the Wnt-signaling pathway (*β-catenin*, *lef1*, and *wnt5*) are expressed early in the wound epidermis during wound healing. By 24 hpa, *lef1* and *wnt5* are restricted to the basal layer of the wound epidermis, while *β-catenin* expression is maintained in the wound epidermis[13]. In addition, the basal layer of the wound epidermis expresses *bmp2b*, *shh*, and *patched1*[15]. The homeobox transcription factors *msxA* and *msxD* are expressed in the wound epidermis starting at 12–24 hpa[14,15]. In newt, it was shown that the AEC is essential for regeneration and its removal halts regeneration until a new wound epidermis is formed[16,17]. Between 12 and 24 hpa, the second phase of regeneration, blastema formation, is initiated. The blastema, a group of progenitor-like cells, is believed to derive from dedifferentiated mesenchymal cells proximal to the amputation plane. The zebrafish blastema is characterized by the expression of two *msx* genes (*msxB* and msx*C*), transcriptional repressors that presumably maintain the dedifferentiated stage of blastemal cells[14,18]. Little is known about the transcriptional regulation of the *msx* genes in the blastema. Blocking Fgf signaling, using a pharmacological inhibitor for Fgf receptor 1, abolishes *msxB*/*C* expression and halts fin regeneration[19]. Furthermore, bone morphogenic protein 4 (*bmp4*), homeobox transcription factors *hoxA11B* and *hoxA13B*, and the retinoic acid receptor-γ (*rar-γ*) are also expressed in the blastema at various time points[20,21,22]. The final phase of fin regeneration is termed "regenerative outgrowth" (24 hpa until completion of regeneration). The actual regrowth of the fin-tissue takes place during this phase. The blastema subdivides into a distal and a proximal part, with the slowly proliferating distal blastema maintaining *msxB/C* expression and progenitor-like cells[23]. Over the course of 1–2 weeks, the size and shape of the original fin will be restored, with growth peaking at 72 hpa[24].

Zebrafish fin regeneration has been studied for over a decade using expression analysis of candidate genes[7], temperature-sensitive mutangenesis screens[25], differential display methodology[26], and other technologies. In Medaka, another teleost fish, a small-scale cDNA microarray screen was reported using 2,900 ESTs, which shared no homology to known genes[27]. Here we describe a large-scale microarray screen using Affymetrix arrays containing approximately 15,000 zebrafish transcripts. We identified 829 differentially expressed transcripts during fin regeneration and established a comprehensive database containing information about expression, functional classification, and current knowledge from the literature of each of those transcripts. To verify the validity of our microarray screen, we performed RT-PCR and *in situ* hybridization on a subset of those transcripts. Furthermore, we provide evidence for the activation of the Bmp signaling pathway during regeneration. In conclusion, we hope that this work will provide a novel and comprehensive information database for fin regeneration.

## MATERIAL AND METHODS

### Zebrafish Husbandry and Fin Amputations

AB wild type zebrafish were maintained as previously described[8]. All experiments were performed using 10- to 18-month-old, adult, male and female zebrafish. For caudal fin amputations, fish were anesthetized in 0.1% tricaine and fins were cut using razor blades. Directly after amputation, fish were shifted to 33°C water for the remainder of the experiment. Uncut control fish were shifted to 33°C in parallel.

### Microarray Analysis

The two experimental sets were derived from groups of independently amputated fish. Total RNA was isolated from 15–20 fins per time point (uncut as control and 12, 24, 48, and 72 hpa) and per experiment using Trizol™ (Invitrogen); 2 µg of total RNA were biotin labeled using Gene Chip One-Cycle Target Labeling (Affymetrix) and hybridized to Affymetrix zebrafish genome arrays according to manufacturer's guidelines (Affymetrix). All microarray experiments were performed in duplicate. Data were normalized using overall intensity. Only transcripts with expression levels higher than 1000 and "present call" in both experimental sets, at least at one time point, were considered. Of those, transcripts with greater than 2.5 fold up- or down-regulation compared to uncut control were identified. Furthermore, for down-regulated transcripts, the control samples had to be above 1000 and "present call" based on the assumption that a down-regulated gene had to be expressed in the fin before amputation. For differentially regulated transcripts, we searched the following databases for homologies: the UniGene database, the "nonredundant" NCBI database, and the "monthly" NCBI database. Genes with homologies less then 50% were labeled "weakly similar", 50–70% homology were labeled "moderately", 70–85% homology were labeled "strongly", and genes with over 85% homology were labeled "highly similar". Hierarchical clustering analysis was performed using "Cluster" and "Treeview" software[28].

### Semi-Quantitative RT-PCR

For RT-PCR analysis, first-strand cDNA was generated using $dT_{20}$ primers and Superscript™ III Reverse Transcriptase (Invitrogen). The PCR conditions and primers are shown below. To ascertain the specificity of the indicated primers, we sequenced the amplified PCR samples.

| Gene | forward (5′) primer | reverse (3′) primer | ann. | size |
|---|---|---|---|---|
| Jagged1a | AATGAAGAAATTCGCTGGCGGT | ACGGCAGTAGTAAAACATAGGC | 57°C | 680bp |
| Sox11A | GGCACTTCTTCCCTTTATCATT | TTCAGCCAGACCACCGACATTC | 50°C | 709bp |
| MdkA | gcgCGGGGTATAAAAGTAGCA | gcGAAGGGCAAAGTCAACGATT | 50°C | 603bp |
| SallI | gcgGAGCAGATTCGTCACCAGATT | gcgGGGGCTGAGGTAGAAGGGTAT | 56°C | 439bp |
| dlx5A | AACCATTTACTCCAGTTTCCAG | GGTAGAGGAATACCACGAAGAG | 50°C | 351bp |
| bambi | ACTCAGAGCTTCTTTGTTAGGG | ACCTTATCTGTACCCTGGTGTC | 57°C | 392bp |
| dact2 | gcgGAAAGGTAGCGATGGTAAGGA | gcgTGCTTGCTGCGATAGTTGA | 56°C | 233bp |
| GDF6A | gcgACCGTCCCCATCAGGCGTCT | gcgGGCTCTTTCTTGCTGCGTCCG | 56°C | 256bp |
| Her6 | gcgCACAAATGACCGCTGCCCTAA | gcgTCAAAAAGGCGAACTGTCCGT | 56°C | 399bp |
| BMP8A | gcgGGGACTGAGTTTGGTTGGAGC | gcgAGGTTATGGGTGGGTCTGGTG | 54°C | 429bp |
| Sox11B | gcgGTGGTTATTCTGTGGGGTTGC | gcgTGCTTCCTGCCATACCTGTG | 54°C | 299bp |
| BMP3 | gcgCAAGTGAAGAAGAGCGAGCAG | gcgGGTGCCCTCTGGAGTTGAT | 56°C | 487bp |
| TGFβRII | gcgAACCGATGTCTACTCTATGGC | gcgGTCCAGGTCGTCGTCC | 56°C | 307bp |
| β-actin | TTCACCACCACAGCCGAAAGA | TACCGCAAGATTCCATACCCA | 57°C | 223bp |

All sequences are in 5' to 3' orientation.
ann.= annealing temperature for PCR

#### *In situ* Hybridization and Antibody Staining

*In situ* hybridization probes for *bambi* and *her6* were *in vitro* transcribed from purified RT-PCR fragments:

> *bambi* — forward (5') primer: 5'-gcgACTTTGTGCGATGGCTGTT-3', reverse (3') primer: 5'-gcgtaatacgactcactatagggagaCAGACTGCGACCTTACCACTT-3';
> *her6* — forward (5') primer: 5'-gcgACACTACACCTGATAAACCCA-3', reverse (3') primer: 5'-gcgtaatacgactcactatagggagaATGAACTTCTCTGCCTTTTG-3'.

The *dlx5A* probe was generated using the full-length coding sequence cloned by RT-PCR:

> forward (5') primer: 5'-gcgATCGATgccaccATGACTGGAGTATTCGACAGA-3', reverse (3') primer: 5'-GCGtctagaTCAAGGCACCATTCAGA-3'.

This was subcloned into a modified version of pCS2 containing the following T7 promoter sequence: 5'-TAATACGACTCACTATA-3". All three probes were transcribed *in vitro* using T7 RNA polymerase and DIG RNA-labeling mix (Roche). Whole-mount *in situ* hybridization was performed as previously described[19]. To control for the specificity of the staining, we performed *in situ* hybridization using a probe against *gfp* as negative control, resulting in no staining. For further analysis, whole-mount *in situ* hybridizations were cryosectioned. For phospho-Smad 1/5/8 stainings, a rabbit polyclonal antibody (Cell Signaling Technology) was used. Fins were fixed in 4% PFA overnight at 4°C, cryosectioned, and stained as previously described[29].

### RESULTS

#### Experimental Design of Microarray Screen

In order to study the molecular mechanisms of fin regeneration, we performed microarray analysis. Total RNA from regenerating caudal fins (Fig. 1A) was labeled and hybridized to Affymetrix "zebrafish genome arrays" containing approximately 15,000 transcripts. Amputated fish, as well as uncut control fish, were maintained at 33°C to accelerate regeneration. We analyzed four different time points during regeneration: 12, 24, 48 and 72 hpa. Data were normalized and genes with a consistent (i.e., in both experimental sets) up- or down-regulation ≥2.5 fold compared to uncut, at least at one time point, were identified. For control purposes, we assessed expression changes of 15 genes that are known to be up-regulated during fin regeneration (Fig. 1B). All of those genes were up-regulated on our microarrays during regeneration.

#### Statistical Analysis, Annotation, and Verification of Microarray Data

A total of 829 transcripts were differentially expressed ≥2.5 fold at one or more time points during regeneration. Of those, 563 (68%) were up-regulated, while only 266 (32%) were down-regulated, indicating that regeneration is predominantly an active process involving complex regulation and altered gene expression (Table 1, total numbers). To quantify the reproducibility of our experiment, we examined how often a single transcript at a single time point showed conflicting results in the two experimental datasets (e.g., a transcript was up-regulated in the first experimental set at a given time point and down-regulated in the second experimental set at the same time point, or vice versa). In our view, microarray experiments provide mainly qualitative data (up- or down- regulation of a transcript compared to its control) and only to a lesser degree quantitative data (fold up- or down-regulation). Therefore, we only compared the qualitative result of each data point and not its exact fold change. For this purpose, we defined a transcript differentially expressed ≥1.5 fold over uncut, as up- or down-regulated. Of the 3316

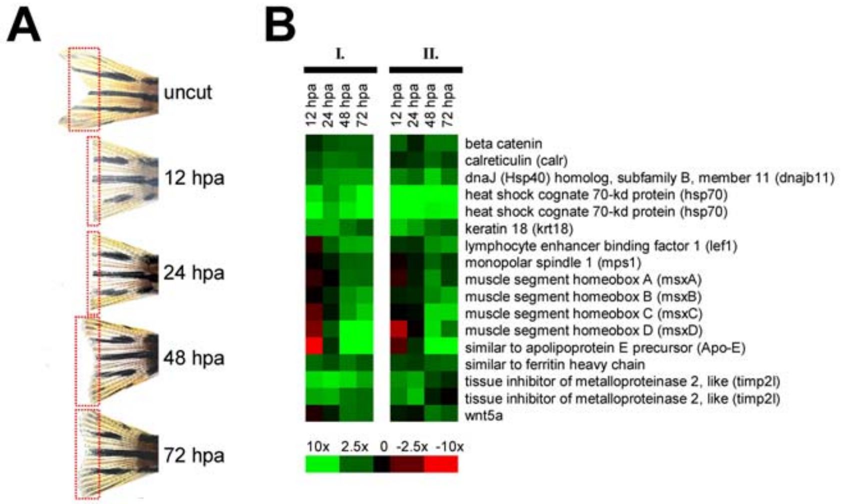

**FIGURE 1.** Experimental design of microarray screen and expression pattern of control genes. (A) Uncut fins and fins 12, 24, 48, and 72 hpa where subjected to microarray analysis. Red boxes demarcate the regions of caudal fins used for isolation of RNA. (B) Expression pattern of 15 genes, which are known to be up-regulated during fin regeneration. Green color indicates an increase of mRNA compared to uncut fins and red color indicates a decrease. The microarray analysis was performed in duplicate. The left column represents the first experimental set (I.) and the right column the second experimental set (II.). Note that all 15 genes, which were previously known to be up-regulated during fin regeneration, were also up-regulated in our screen (B).

**TABLE 1**
**Categories of Transcripts Differentially Regulated and the Number of Genes in Each Functional Group**

| Function | up | down |
|---:|---|---|
| Apoptosis | 4 | 7 |
| Cell-cycle | 25 | 6 |
| Cell-mobility | 21 | 7 |
| Cytoskeleton | 35 | 10 |
| ECM | 49 | 13 |
| Immune-response | 18 | 16 |
| Metabolism/Transport | 134 | 60 |
| Proliferation | 11 | 8 |
| Signaling | 67 | 38 |
| Stress-response | 33 | 1 |
| Transcription | 55 | 18 |
| Unknown | 140 | 91 |
| **Total** | **563** | **266** |

data points analyzed (829 transcripts multiplied by 4 time points), only 26 (0.78%) showed this incongruity of regulation in the two experimental sets, demonstrating that the majority of our data points are reproducible.

First, we looked at the expression changes over time. To do so, we performed hierarchical clustering analysis on the differentially expressed genes (Table S1). We observed that 87 (10.5%) transcripts showed the strongest differential expression at early time points (12 or 24 hpa), 292 (35.2%) showed the strongest differential expression at late time points (48 or 72 hpa), and the remaining 450 (54.3%) were consistently up- or down-regulated throughout all time points (Tables 2 and S1).

**TABLE 2**
**Numbers of Genes in Each Temporal Expression Pattern Cluster**

| Cluster | up | down |
|---|---|---|
| early regulated | 35 | 52 |
| always regulated | 288 | 162 |
| late regulated | 249 | 43 |

Next, we analyzed the 829 differentially expressed transcripts for homology to known genes by using their GenBank entry as query to search for homologs in the UniGene database, the "nonredundant" NCBI database, and the "monthly" NCBI database. Of the 829 differentially expressed transcripts, 695 (84%) demonstrated homology to a known gene or cDNA (Fig. 2); 63 (7.5%) showed no significant homology to known genes or cDNAs, but their genomic loci were identified; and the remaining 71 (8.5%) lacked any homology to genes, cDNAs, or genomic sequences.

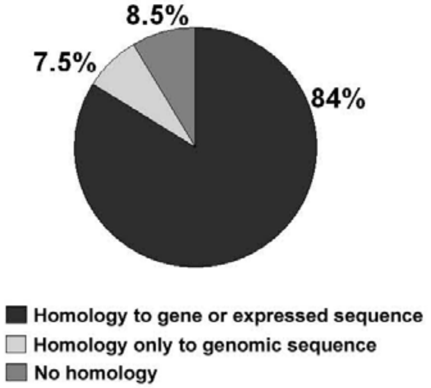

**FIGURE 2.** Of the differentially expressed transcripts, 91.5% shared similarity with known genes; 695 of the 829 differentially expressed transcripts (84%) were similar to known genes; 63 (7.5%) showed homology only to genomic sequences, but not to known genes; 71 (8.5%) contained no significant homology to cDNAs or genomic sequences.

To ascertain the validity of our microarray study, we analyzed selected genes by semi-quantitative RT-PCR. We performed RT-PCR for genes up-regulated early and throughout regeneration (Fig. 3A: *jagged1a*, *sox11A*, and *mdkA*), for genes up-regulated at later stages only (Fig. 3B: *sall1*, *dlx5A*, *bambi*, *dact2*, *gdf6A*, *her6*, *bmp8A*, and *sox11B*), and for down-regulated genes (Fig. 3C: *bmp3* and *tgfβ receptor II*). Expression patterns determined by semi-quantitative RT-PCR correlated well with our microarray data (Fig. 3, left and right panel) demonstrating the validity of our screen.

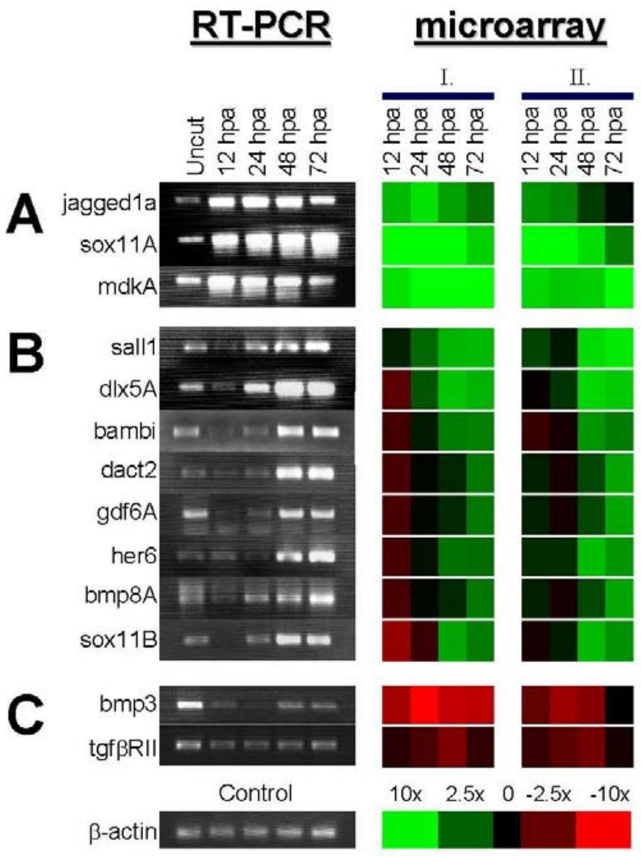

**FIGURE 3**. Verification of microarray results by RT-PCR. Semi-quantitative RT-PCR was performed on selected genes using uninjured or regenerating fin-tissue isolated at 12, 24, 48, and 72 hpa as template. Genes were grouped for early up-regulation (A), late up-regulation (B), or down-regulation (C). Note that RT-PCR results (left panel) correlate well with microarray results (right panel).

### Generation of a Gene Database for Fin Regeneration

Next, we looked at the function of transcripts with homology to known genes. Whenever possible, we assigned the identified genes into one of the following functional categories: apoptosis, cell-cycle, cell-mobility, cytoskeleton, extracellular matrix (ECM), immune-response, metabolism/transport, proliferation, signaling, stress-response, and transcription (Table 1). If a gene was involved in several functional categories, we would assign it to multiple categories. Most genes (194) were attributed to the metabolism and transport category. The second and third largest groups were signaling genes (105) and transcription factors (73), respectively. Interestingly, regeneration also induced expression of 33 transcripts, known to be involved in stress response, including 12 heat shock proteins (Hsp). *hsp40*, *hsp60*, and *hsp70* have been implicated in fin regeneration before[30,31]. Since control as well as regenerating fish were maintained in the same water temperature, this indicates that those genes were not up-regulated by temperature, but through the amputation-regeneration process. In addition to categorizing the genes, we also annotated 464 of them with brief descriptions about their background and potential function based on current knowledge from the literature (Table S1). Altogether, we established a comprehensive and searchable database of fin regeneration genes.

### Regulators of Wound Healing are Up-Regulated at 12 hpa

We hierarchically clustered the categories of signaling and transcription (Figs. 4A, 5A). The majority of those genes were up-regulated during fin regeneration. We further subdivided them according to their temporal expression. For both groups, most genes exhibited either a consistent up-regulation throughout regeneration (Figs. 4C, 5C), or a late up-regulation (Figs. 4D, 5D). Only five genes were up-regulated early, primarily at 12 and 24 hpa (Figs. 4B, 5B). At 12 hpa, wound healing takes place. Epidermal cells adjacent to the wound migrate and cover the stump, initially without undergoing proliferation. Those five early genes include three signaling factors (Fig. 4B: a homolog of *hb-egf*, *activin βA*, and *jagged1a*) and two transcription factors (Fig. 5B: *atf3* and a homolog of *cbp/p300-interacting transactivator 3*). Interestingly, it was shown in mice that HB-Egf is important for wound healing by accelerating keratinocyte migration, rather than effecting proliferation[32]. Furthermore, Atf3 was shown to be specifically expressed in leading-edge keratinocytes, the first row of cells closing the gap of a healing skin wound[33]. Lastly, Activin has been shown to fulfill positive regulatory functions during wound healing[34,35]. Taken together, these data not only verify once more the validity of our microarray screen, but also advocate the evolutionary conservation of wound healing mechanisms.

### Activity of Signaling Pathways During Regeneration

Signaling factors are the second largest category of genes regulated during regeneration. Previous evidence suggested the involvement of the major developmental signaling pathways in fin regeneration, including the Bmp, Fgf, Notch, retinoic acid, Shh, and Wnt pathway[13,15,21,24,36]. In our screen, we found evidence for the induction of all of those pathways. Table 3 provides a summary of the differentially regulated genes in each pathway. All the changes observed are relative in respect to uninjured fins. Hence, if a signaling pathway is already active before injury and down-regulated during regeneration, it can still be active, but simply to a lesser degree. The Bmp, Fgf, and Notch pathways have predominantly increased expression of positive regulators and target gene expression is induced, indicating that these pathways are activated during regeneration. In contrast, up-regulation of the negative regulator *tgif*[37] and down-regulation of the positive regulator *raldh14* suggest that the activity of the retinoic acid pathway is reduced. Nevertheless, five known target genes of retinoic acid are induced. The target gene *hgfl* is down-regulated, but this gene is the only one in the group, whereupon retinoic acid signaling acts as a repressor[38]. In addition to the retinoic acid pathway, Tgf-β signaling appears to be diminished as well. Four negative regulators of Tgf-β are up-regulated during regeneration, while three positive regulators showed reduced expression (Table 3). Nevertheless, several known Tgf-β target genes are up-regulated. Hence, retinoic acid and Tgf-β signaling appear reduced even though expression of their respective target genes was increased. To explain these results, it should be emphasized that a microarray screen is restricted to the transcripts that are present on the array. Furthermore, post-transcriptional regulations, which cannot be studied by DNA microarrays, contribute to the regulation of signaling pathways. Therefore, other positive regulators of retinoic acid and Tgf-β signaling might be activated during regeneration. Signaling pathways are often subjected to tight feedback regulation and stimulation of a pathway results in expression of negative regulators. For example, in the Wnt pathway, expression of *β-catenin*, a downstream effector of canonical Wnt signaling, induces expression of *axin2*, which acts as a negative regulator on β-Catenin[39,40,41]. Both, *β-catenin* and *axin2*, are also up-regulated in our screen. In conclusion, our microarray screen provides numerous new insights into signaling pathways during fin regeneration, although further experiments are needed to establish the exact spatial and temporal activity of each pathway.

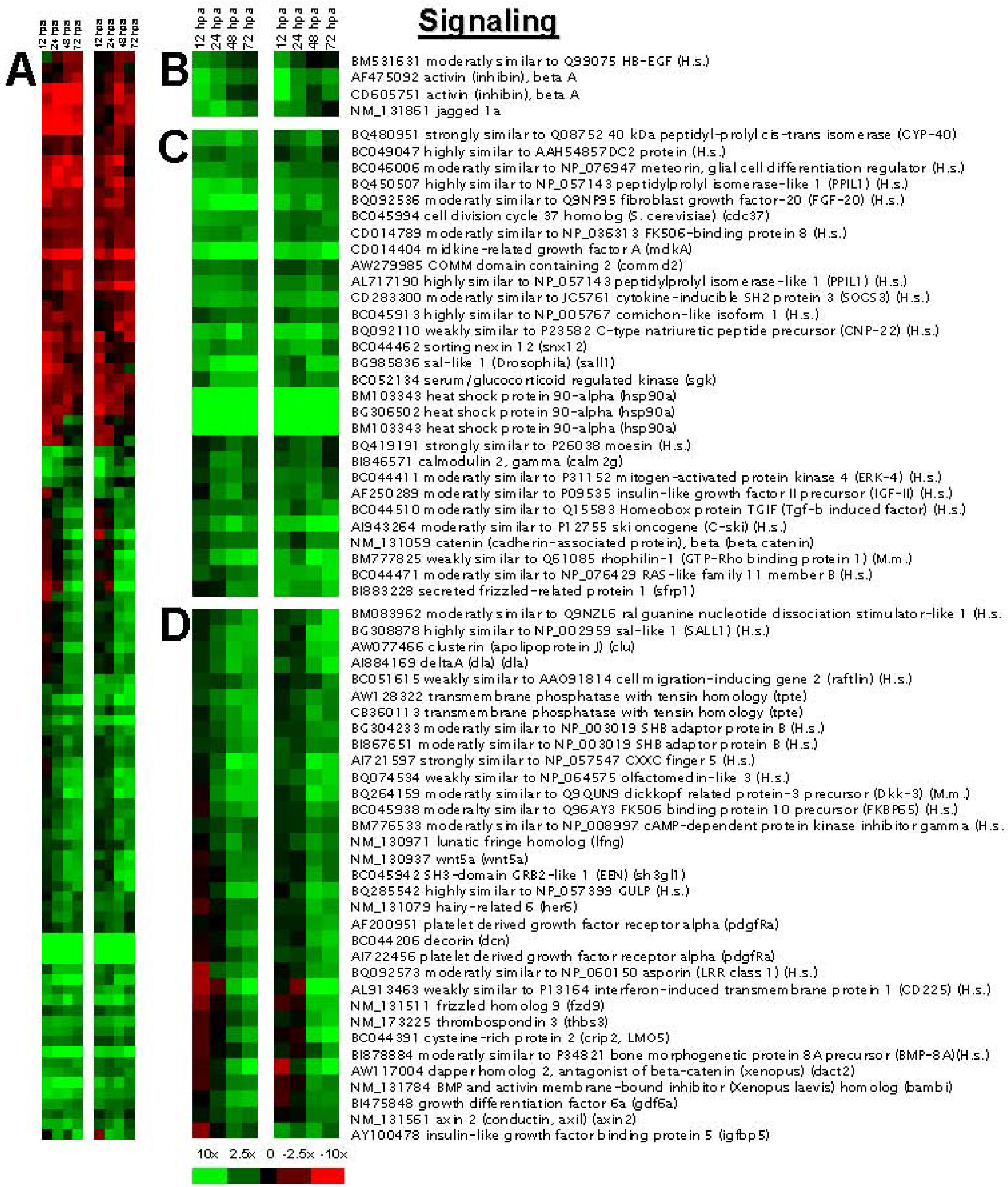

**FIGURE 4.** Expression patterns of genes involved in signaling. (A) Hierarchical clustering of all signaling genes. (B–D) Expression patterns, GenBank accession numbers, and names of signaling genes, which showed increased expression early (12 and 24 hpa, B), later (48 and 72 hpa, D), or throughout regeneration (C). H.s., Homo sapiens; M.m., Mus musculus.

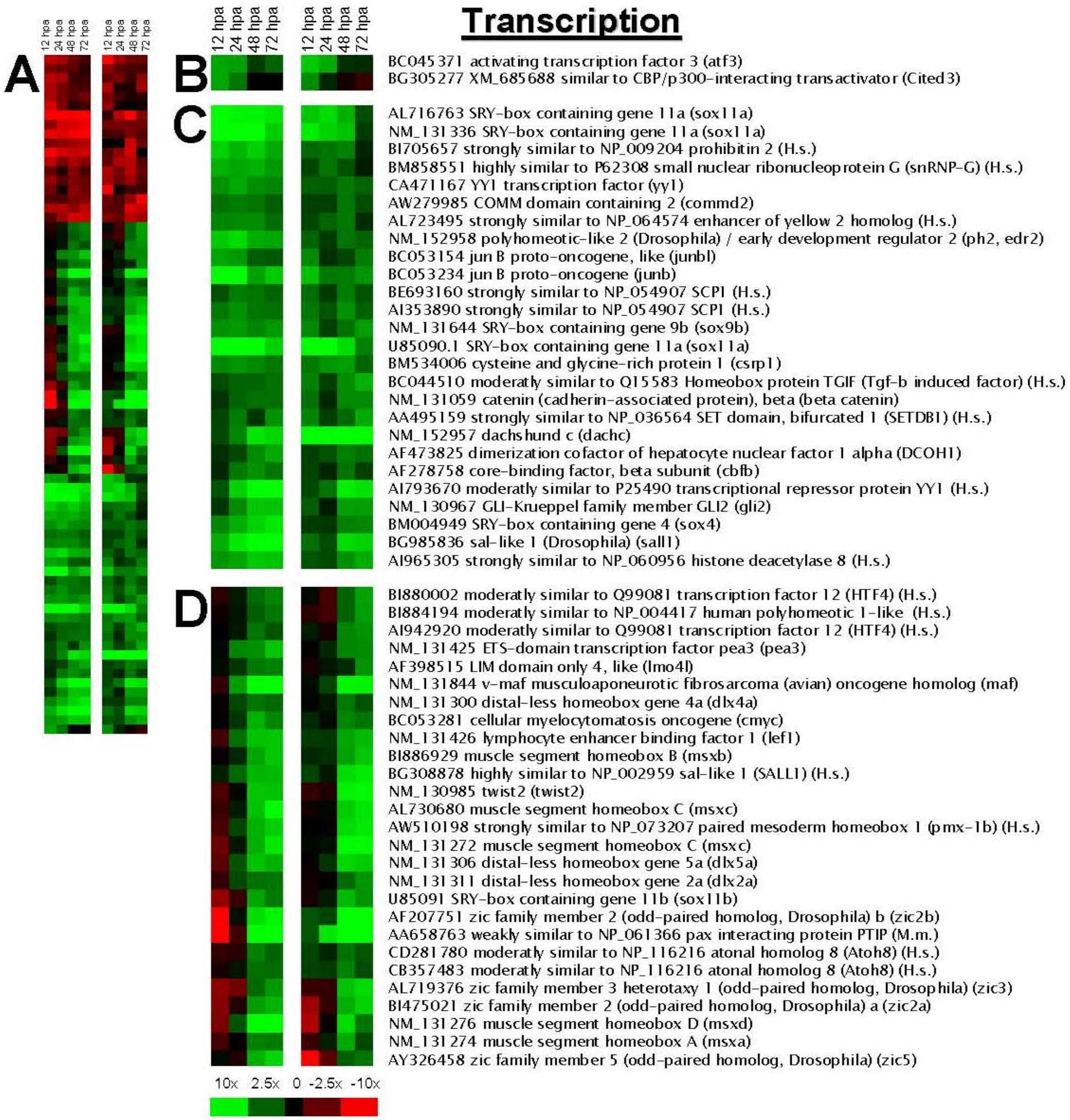

**FIGURE 5**. Expression patterns of genes involved in transcription. (A) Hierarchical clustering of all transcription factors or genes involved in transcription. (B–D) Expression patterns, GenBank accession numbers, and names of transcription genes, which showed increased expression early (12 and 24 hpa, B), late (48 and 72 hpa, D), or throughout regeneration (C). H.s., Homo sapiens; M.m., Mus musculus.

### dlx5A is Expressed in the Basal Wound Epidermis

To further validate the results of our screen, we performed *in situ* hybridization of three up-regulated genes. One of them is the "distal-less-related", DNA-binding, homeobox transcription factor Dlx5A. We chose to look at this gene because of evidence that *dlx* genes can regulate *msx* expression[42,43]. *dlx5A* is already expressed at 24 hpa in the basal layer of the wound epidermis (Fig. 6). Furthermore, by 72 hpa, the expression in the basal wound epidermis is maintained in the distal region of the regenerate. There appears to be an expression gradient from distal to proximal, with the highest expression a few cell diameters further

**TABLE 3**
**Summery of Signaling Molecules Differentially Expressed During Fin Regeneration***

| Pathway | Regulators | | Target genes | |
|---|---|---|---|---|
| | up | down | up | down |
| **Bmp** | *bmp8a* (→)<br>*gdf6* (→)<br>*bambi* (⊣) | *bmp3* (⊣) | *bambi*<br>*dlx2A*<br>*dlx5A*<br>*periostin* | |
| **Fgf** | *fgf20* (→)<br>*shh* (⇄) | | *pea3*<br>*ph2*<br>*dlx2A*<br>*dlx5A* | |
| **Notch** | *jagged 1a* (→)<br>*deltaA* (→)<br>*lunatic fringe* (⇄) | | *Her6* | |
| **Retinoic acid** | *tgif* (⊣) | *raldh 14* (→) | *map7*<br>*rai14*<br>*cx43*<br>*mdkA*<br>*hph1* | *hgfl* |
| **Shh** | | | *sall1* | |
| **Tgf-β** | *ski* (⊣)<br>*bambi* (⊣)<br>*asporin* (⊣)<br>*tgif* (⊣) | *tgf-βRII* (→)<br>*ltbp-1* (→)<br>*runx3* (→) | *fstl1*<br>*integrin α5*<br>*integrin β5*<br>*tgfbi*<br>*periostin* | *tieg2*<br>*dspp*<br>*tgfbi* |
| **Wnt** | *wnt5A* (→)<br>*β-catenin* (→)<br>*frizzled 9* (→)<br>*gli2* (→)<br>*sall1* (→)<br>*dkk-3* (⊣)<br>*cxxc5* (⊣)<br>*secreted frizzled 1* (⊣)<br>*N-cadherin* (⇄)<br>*axin2* (⊣) | *frizzled 9* (→)<br>*sox3* (⊣) | *survivin*<br>*fgf20*<br>*sox9*<br>*fibronectin*<br>*cx43*<br>*periostin*<br>*bambi*<br>*zic3*<br>*dapper2*<br>*axin2* | *enc1*<br>*zic3*<br>*dapper2* |

(→) indicates a positive regulator
(⊣) indicates a negative regulator
(⇄) indicates positive and negative regulation

* Genes that are in both the up and down column did show up- and down-regulation at different time points during the observed 72-h time frame.

proximal of the distal tip of the fin. In particular, whole mount *in situ* hybridization showed these spots of most intense expression in the more distal areas of the regenerates at 72 hpa (Fig. 6A). This expression is similar to the expression of *shh* at 72 hpa, which is dependent on Fgf-receptor signaling, since pharmacological inhibition of the Fgf receptor abolishes *shh* expression[15,19]. Interestingly, *dlx* expression is also known to be downstream of Fgf. Choi et al. demonstrated that during bone formation in mice, Fgf2 induces Bmp2, an upstream regulator of *dlx5* and *msx2*[43]. Moreover, Cheng et al. showed in chicken that Dlx5 is a transcriptional regulator of *msx2* expression in the limb AER[42]. During fin regeneration, at least

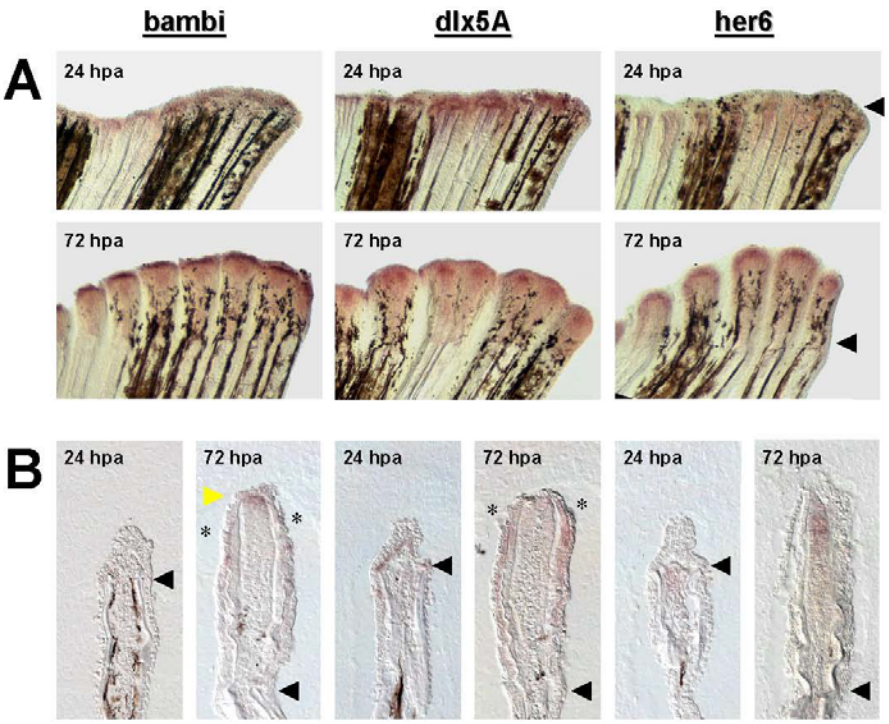

**FIGURE 6.** *bambi*, *dlx5A*, and h*er6* are up-regulated during regeneration. (A) Expression patterns at 24 and 72 hpa were analyzed by whole mount *in situ* hybridization (red signal). *bambi* and *her6* are weakly expressed at 24 hpa and strongly at 72 hpa. *dlx5A* is already expressed at 24 hpa and maintained by 72 hpa. (B) Longitudinal sections of *in situ* hybridization. Yellow arrowhead indicates expression of *bambi* in the distal blastema. Asterisks point out gap in expression of *bambi* and *dlx5A* along the basal layer of the wound epidermis. Black arrowheads indicate amputation plane.

four msx genes are expressed (*msxA*, *B*, *C*, and *D*). Based on homology comparison, it was speculated that mammalian Msx1 is the homolog of MsxB and C in the fish, while Msx2 is the homolog of MsxA and D[14]. Indeed, *msxA* and *D* are expressed in the basal layer of the wound epidermis, similar to *dlx5A*[14]. Future experiments will show if there is a direct transcriptional regulation of *msxA* and *D* by Dlx5A during regeneration. In summary, these results indicate a possible transcriptional cascade, containing Fgf signaling, *dlx5A* expression, and *msxA*, *D* expression.

### her6, a Downstream Target of Notch Signaling, is Expressed in the Mesenchyme During Blastema Formation and in the Proximal Blastema

Notch signaling is an evolutionarily conserved pathway, critical for tissue morphogenesis during development and tissue maintenance and repair in the adult. The zebrafish *her6* gene encodes a basic helix-loop-helix transcription factor, from the hairy/enhancer-of-split family (Hes)[44]. Hes genes are under direct transcriptional control of Notch signaling and, therefore, are valuable reporters of Notch activity[45]. Two ligands of the Notch receptor were up-regulated in our microarray experiments during fin regeneration: *jagged 1a* and *deltaA* (Fig. 3A). Furthermore, expression of the modulator *lunatic fringe* was induced. Previous data demonstrated that *Notch 1b* and *deltaC* are also expressed during fin regeneration[36]. Therefore, we looked at the spatial expression of the downsteam effector *her6* to assess where the Notch signal is translated into transcriptional readout. At 24 hpa, when the blastema forms, *her6* was weakly, but broadly, expressed in the mesenchyme (Fig. 6). During regenerative outgrowth, at 72 hpa, *her6* is strongly expressed in the proximal blastema (Fig. 6B). Notch activity is often connected with maintaining cells in a progenitor-like stage[46]. Because the proximal blastema fulfills a transitional role between the stem cell–like distal blastema and the newly formed, differentiated proximal fin tissue, we hypothesize that Notch signaling during regenerative outgrowth could be involved in maintaining a progenitor-like stage of proximal blastema cells. Since *her6* is also expressed broadly at 24 hpa in the mesenchyme, this would also suggest a possible role for Notch signaling in the dedifferentiation/ blastema-formation process.

### Smad 1/5/8 is Phosphorylated in the Mesenchyme and in the Basal Layer of the Wound Epidermis

The third gene we analyzed in more detail was *bambi* (also called *nma*), a negative feedback regulator of Bmp signaling[47,48,49]. Bambi is a naturally occurring, dominant, negative receptor of the Tgf-beta type I receptor family. Expression of *bambi* itself is regulated by Bmp and Wnt signaling[50,51]. During fin regeneration, *bambi* is very weakly expressed at 24 hpa (Fig. 6). By 72 hpa, expression is strong in the distal blastema and in the basal layer of the wound epidermis. Again, the peak of expression in the basal layer of the wound epidermis is further proximal from the far distal end of the regenerate (Fig. 6B). Since *bambi* expression can be regulated by Bmp and Bambi is also a negative repressor of Bmp signaling, we analyzed the activity of this pathway during fin regeneration. In addition to *bambi*, *bmp3*, a regulator of osteogenic Bmps[52], was down-regulated, while *bmp8A*, a positive regulator, was induced during regeneration (Figs. 2B, C). To determine the status of Bmp signaling during fin regeneration, we looked at downstream-effector molecules. Bmp activity results in phosphorylation of members of the 1/5/8 family of Smad proteins and their accumulation in the nuclei. Using an antibody specific for phosphorylated serine in the C-terminal SSXS motif of Smad 1/5/8 proteins, we observed that Bmp signaling is active in the mesenchyme proximal to the amputation plane at 24 hpa (Fig. 7A). In addition, cells of the basal layer of the wound epidermis are positive for phosphorylated Smad 1/5/8. During the outgrowth phase (72 hpa), phosphorylation of mesenchymal cells is reduced and sporadic throughout the regenerate (Fig. 7B). Cells of the basal layer of the wound epidermis in the distal half of the regenerate are also positive, but exhibit an area of reduced Bmp activity (Fig. 7B asterisks). Since *bambi* expression is highest in this area of the basal layer of the wound epidermis and Bambi is an established repressor of Bmp signaling in zebrafish as well as other species[49], we conclude that *bambi* expression contributes to the regulation of Bmp signaling in the wound epidermis. In addition to the epidermal expression, Bmp signaling appears very active at 24 hpa in the mesenchyme undergoing disorganization and giving rise to the blastema (Fig. 7A). *bambi* expression is not detectable in the mesenchyme at 24 hpa, but by 72 hpa, it is strongly expressed in the distal blastema (Fig. 6B). Due to the negative feedback loop of Bambi-Bmp, it is plausible that Bmp activity induces *bambi* expression, thereby fine-tuning the Bmp signal. Thus, Bmp signaling is activated during regeneration and potentially modulated by *bambi* expression.

### DISCUSSION

Fin regeneration in zebrafish is an important model system for regeneration of complex body structures. Nevertheless, fundamental questions like blastema formation or whether regeneration is a recapitulation of embryonic development are still largely unknown. The aim of this study was to generate a comprehensive and detailed database of differentially expressed genes during zebrafish caudal fin regeneration. We generated expression profiles of approximately 15,000 zebrafish transcripts at 12, 24, 48, and 72 hpa. Based on this screen, we constructed a database of 829 differentially expressed transcripts and validated our results in three ways. First, we demonstrated that 15 genes that are known from the literature to be up-regulated during regeneration are also up-regulated in our database. Second, we validated the expression of 13 genes from our database, which had previously not been implicated in fin regeneration, by RT-PCR. Third, we performed *in situ* hybridization for 3 genes, to confirm the RT-PCR

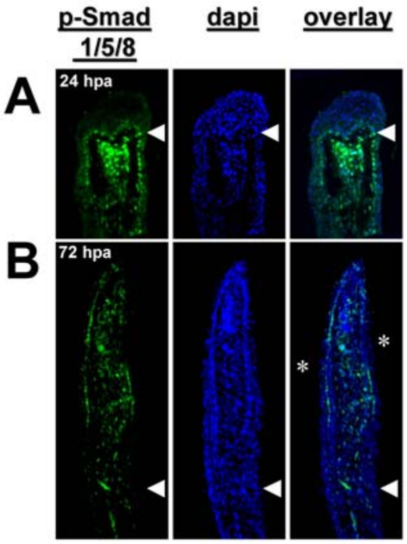

**FIGURE 7**. Smad 1/5/8 is phosphorylated in the mesenchyme and in the basal layer of the wound epidermis. (A) 24-hpa Smad 1/5/8 is phosophorylated in the mesenchyme proximal to the amputation plane and in the basal wound epidermis. (B) At 72 hpa, Smad 1/5/8 is still phosphorylated in the wound epidermis, but reduced in the area indicated with asterisks. White arrowheads indicate amputation plane.

results and to further analyze their spatial expression patterns during caudal fin regeneration. Our goal was to go beyond simply listing genes. Thus, we functionally categorized most of those 829 genes and annotated 464 genes with brief comments about their potential function based on current knowledge from the literature. To our knowledge, this is the largest gene expression screen for fin regeneration.

Our current understanding of fin regeneration is mainly based on candidate gene approaches. In addition, two large-scale analyses have been published. Padhi et al. performed SSH and DDRT-PCR screens with zebrafish caudal fins 24 and 96 hpa, isolating 153 known and 145 unknown genes that were up-regulated during regeneration[26]. Katogi et al. generated cDNA libraries from Medaka caudal fins at 72 and 240 hpa and screened 2900 unknown ESTs by microarray technology, identifying 52 up-regulated ESTs (1.8% of the spotted ESTs)[27]. Comparison of the data of Padhi et al. with our results showed that 24 of the known genes were also differentially expressed in our experiments despite the fact that different time points were analyzed. In contrast, the screen of Katogi et al. could not be compared to our results, as this screen was done in Medaka and focused on ESTs without known homologies. Those screens were based on SSH and DDRT-PCR, as well as generation of cDNA libraries, respectively. Such technologies tend to have a bias towards highly expressed genes. The recent availability of Affymetrix microarrays for zebrafish provided the possibility to perform a large-scale expression screen of 15,000 transcripts, covering roughly one-third of the estimated zebrafish genome. One critical parameter of microarray screens is the fold-induction cut-off. In the presented work, we use 2.5 fold induction/repression as a minimum. This was based on induction values of known genes, up-regulated during regeneration. We identified 829 differentially expressed transcripts. Less stringent parameters would allow the generation of even more complex databases. Thus, our microarray screen is an important addition to the previous research, establishing a comprehensive collection of genes involved in fin regeneration.

Furthermore, we provide transcription profiles of down-regulated genes, which have been largely disregarded in previous analysis. Regeneration is believed to depend on dedifferentiation processes. From other systems, it is known that cells can be actively maintained in their differentiated state and inhibition of those mechanisms is essential for initiating regeneration[53]. For that reason, down-regulation of such “differentiation genes” is an important, yet neglected, aspect of regeneration. Our work could provide a new foundation to open up this field of research.

Investigating early regulators of regeneration, we identified three genes previously implicated in mammalian wound healing processes. The identification of genes with known importance for mammalian wound healing in our fin regeneration screen provides support for the conservation of those processes throughout evolution. In addition, these data are encouraging evidence for the potential of our screen in particular, and the zebrafish model in general, to identify pharmacological targets of clinically relevance.

We think that our database will provide the foundation to quickly identify new genes and pathways involved in regeneration and wound healing. In addition, new marker genes for regenerative processes and structures can be established based on our results. Furthermore, new and old zebrafish mutant strains, defective in fin regeneration, could be quickly analyzed for the molecular consequences of their mutations, by comparing their transcriptional profiles with our data. We believe that the generation of this gene expression database will be a useful tool for current and future studies of fin regeneration.

### ACKNOWLEDGMENTS

The authors would like to thank O. Paugois, C. Richards, P. Jhawar, S. Kim, and R. Badakov for excellent fish care; M. Duong for technical support; K. Poss and A. Nechiporuk for protocols; M. Duong, S. Makino, K. Poss, and V. Yin for critical review of the manuscript; and all former and present Keating lab members for helpful discussions. This work was supported by the Austrian Academy of Sciences, the Max Kade Foundation, Inc., and NIH grant IP50HLO74734.
